# Osthole Suppresses Cell Growth of Prostate Cancer by Disrupting Redox Homeostasis, Mitochondrial Function, and Regulation of tiRNA^HisGTG^

**DOI:** 10.3390/antiox13060669

**Published:** 2024-05-30

**Authors:** Jisoo Song, Jiyeon Ham, Gwonhwa Song, Whasun Lim

**Affiliations:** 1 Department of Biological Sciences, College of Science, Sungkyunkwan University, Suwon 16419, Republic of Korea; songjs9251@g.skku.edu; 2 Division of Animal and Dairy Science, College of Agriculture and Life Sciences, Chungnam National University, Daejeon 34134, Republic of Korea; jyham@cnu.ac.kr; 3 Department of Biotechnology, College of Life Sciences and Biotechnology, Korea University, Seoul 02841, Republic of Korea

**Keywords:** prostate cancer, osthole, calcium ion, oxidative stress, tiRNAs

## Abstract

Prostate cancer remains a significant global health concern, posing a substantial threat to men’s well-being. Despite advancements in treatment modalities, the progression of prostate cancer still presents challenges, warranting further exploration of novel therapeutic strategies. In this study, osthole, a natural coumarin derivative, inhibited cell viability in cancer cells but not in the normal prostate cell line. Moreover, osthole disrupted cell cycle progression. Furthermore, osthole reduces mitochondrial respiration with mitochondrial membrane potential (ΔΨm) depolarization and reactive oxygen species (ROS) generation, indicating mitochondrial dysfunction. In particular, osthole-induced ROS generation was reduced by N-acetyl-L-cysteine (NAC) in prostate cancer. In addition, using calcium inhibitors (2-APB and ruthenium red) and endoplasmic reticulum (ER) stress inhibitor (4-PBA), we confirmed that ER stress-induced calcium overload by osthole causes mitochondrial dysfunction. Moreover, we verified that the osthole-induced upregulation of tiRNA^HisGTG^ expression is related to mechanisms that induce permeabilization of the mitochondrial membrane and calcium accumulation. Regarding intracellular signaling, osthole inactivated the PI3K and ERK pathways while activating the expression of the P38, JNK, ER stress, and autophagy-related proteins. In conclusion, the results suggest that osthole can be used as a therapeutic or adjuvant treatment for the management of prostate cancer.

## 1. Introduction

Prostate cancer is the second-leading cause of cancer-related deaths among men in the United States and has the highest incidence rate [1]. The pathogenesis has not yet been well investigated, but hormones like androgens or other environmental factors like age, family history, and obesity may have some connection [2]. Diagnosis is difficult in the early stages because there are no specific symptoms. Although the disease is treated with currently available methods, such as surgical resection, radiation therapy, and hormone therapy, progressive castration resistance or metastasis can frequently occur [3,4]. With the recent increase in systemic therapy use, the prognosis of advanced prostate cancer has improved; however, it still has a high mortality rate [2,5]. Therefore, research is required on management agents and treatments that can effectively manage prostate cancer.

Phytopharmaceuticals are plant-derived compounds with pharmacological effects that are traditionally used to treat diseases [6,7]. Osthole is a coumarin derivative derived from *Cnidium monnieri* and *Angelica pubescens*; it possesses biological effects, such as antitumor, anti-inflammatory, antibacterial, cardiovascular protection, and osteogenesis [8,9]. Previous studies have shown that osthole induces cell cycle arrest in the G2/M phase and regulates the tumor suppressor proteins GNG7 and STAT3, indicating its anticancer effects on breast cancers [10,11,12]. In another study, osthole downregulated the levels of CDC2 and cyclin B1, causing cell cycle arrest, DNA damage, and suppression of migration in liver cancer cells [13]. However, the mechanism by which osthole exerts its anticancer effects on prostate cancer cells has not yet been elucidated.

tiRNAs, which are non-coding RNA, have recently been reported to not only silence RNA but also affect various physiological processes, from cell fate of death or survival to mitochondrial function [14,15]. Pre- or mature tRNAs can be cleaved by the angiogenin (ANG) enzyme to produce tiRNAs, which can be promoted in a sex hormone-dependent manner (e.g., androgens or estrogens) and directly or indirectly affect the survival and death of cancer cells [16,17]. Considering the recent studies on the physiological and possible anticancer effects of tiRNAs, the elucidation of tiRNA mechanisms on anticancer effects will be a powerful tool to understand its pathogenesis and identify therapeutic targets for prostate cancer. These results suggest that tiRNAs have various effects on prostate cancer. However, much research has not yet been conducted. Therefore, we investigated the anti-cancer effect of osthole in prostate cancer.

## 2. Materials and Methods

### 2.1. Reagent

Osthole, also called 7-methoxy-8-(3methyl-2-butenyl) coumarin or 7-methoxy-8-isopentenylcoumarin (Cat No. O9265, Sigma Aldrich, St. Louis, MO, USA), was dissolved in dimethyl sulfoxide. In this study, 0 μM refers to cells treated with DMSO only.

### 2.2. Cell Culture Method

The prostate cancer cell lines PC3 and DU145 and the normal prostate cell line WPMY-1 were purchased from the American Type Culture Collection (Manassas, VA, USA). The cancer cell lines were cultured in RPMI-1640 medium containing 25 mM HEPES supplemented with 10% FBS and 1% penicillin/streptomycin (100 units/mL). These cells were grown until 70% confluent on each dish or plate for appropriate experiments at 37 °C with 5% CO_2_. The cells were then serially incubated in a non-FBS medium for 24 h and further incubated for 48 h with osthole at various concentrations. Furthermore, the normal prostate cell line, WPMY-1, was cultured in Dulbecco’s modified Eagle’s medium supplemented with 5% FBS and 1% penicillin/streptomycin.

### 2.3. Cell Viability Test

The viabilities of PC3, DU145, and WPMY-1 cells were determined using an MTT proliferation kit (Roche, Basel, Switzerland). For the cell viability test, each cancer and normal cell line was seeded in 96-well plates (5 × 10^3^ per well). Subsequently, these cells were treated with osthole from 0 to 50 μM for 48 h. MTT tetrazolium was added to each well and incubated for the next 4 h. Solubilization buffer was added to dissolve the formazan crystals formed by tetrazolium and incubated for sufficient time to dissolve completely. Absorbance was measured at 560 nm and 650 nm using a microspectrophotometer.

### 2.4. Cell Cycle Analysis

For this assay, osthole-treated prostate cells were incubated for 48 h at various concentrations. The cells were collected for fixation and washed serially with 0.1% BSA-PBS. Ethanol in BSA-PBS was used as a fixation buffer. The prepared cells were stained with RNase A and propidium iodide (PI), and the fluorescence of the stained cells was analyzed by flow cytometry as illustrated in a previous study [18].

### 2.5. 3D Hanging Spheroid Formation Assay

For the 3D culture of prostate cancer cells, we used the hanging drop method for spheroid culture. In detail, each 25 μL of cell suspension treated with or without osthole was dropped on the cover of a culture dish and incubated for 48 h for imaging purposes. Changes in spheroid cell morphology were observed using a DM3000 microscope (Leica Microsystems, Wetzlar, Germany), and analyzed using ImageJ software (version 1.8.0).

### 2.6. Quantitative Real-Time PCR Detection

Total RNA was extracted 24 h after osthole treatment. Subsequently, RNA reverse transcription was performed using oligo dT, random primers, and AccuPower premix (Bioneer, Daejeon, Republic of Korea), following the manufacturer’s instructions. Afterward, qRT-PCR was conducted on cDNA using SYBR green, target primers, dNTP, 10× buffer, and Taq polymerase. Fluorescence intensity was analyzed, as reported in a previous study [19].

### 2.7. Detection of ΔΨm Depolarization

Changes in the mitochondrial membrane potential were evaluated by staining the cells with JC-1 dye. JC-1 accumulates in the mitochondria of the healthy cells, leading to red fluorescence, whereas it leads to green in the unhealthy ones. For this assay, prostate cancer cells were treated with various doses of osthole for 48 h. After several washes and staining steps, the relative green/red fluorescence was measured using flow cytometry.

### 2.8. Detection of Reactive Oxygen Species Generation

2′,7′-dichlorofluorescein diacetate (DCF diacetate) is used to stain osthole-treated prostate cancer cells to detect reactive oxygen species (ROS). Prostate cancer cells were treated with osthole with or without NAC (1 mM) for 24 h and then harvested for staining. After several washing steps with PBS, relative fluorescence intensity of 2′,7′-DCF, which is converted depending upon the oxidation by radicals, was then measured using flow cytometry.

### 2.9. Measurement of Calcium Ion Accumulation in Cellular Organelle

The relative levels of calcium ions in prostate cancer cells were measured using Fluo-4 acetoxymethyal (AM) in the cytosol and Rhod-2 AM in the mitochondria. Furthermore, we treated osthole with the specific calcium inhibitors 2-Aminoethoxydiphenyl borate (2-APB) and ruthenium red (RUR) and the ER stress inhibitor 4-Phenylbutyric acid (4-PBA) to trace the suspected calcium ion flow. After treatment with each combination for 48 h, the cells were collected and stained with Fluo-4 AM or Rhod-2 AM dye. After staining, the cells were washed with PBS and analyzed using flow cytometry.

### 2.10. Detection of Mitochondrial Respiration in Prostate Cancer Cells

Mitochondrial respiration in prostate cancer cells was confirmed using Seahorse XFe24 (Agilent Technologies, Santa Clara, CA, USA). To measure various mitochondrial respiration parameters, an XF24 Mito stress kit (Agilent Technology, Santa Clara, CA, USA) was used according to the manufacturer’s instructions. Then, the prostate cancer cells were cultured in 24-well cell culture microplates and treated with 20 μM osthole for 6 h at 37 °C. Finally, oligomycin, FCCP, rotenone, and antimycin A were serially added in real time to the cells in microplates and automatically analyzed using Seahorse Wave Desktop software (version 2.6).

### 2.11. Transfection with Lipofectamine and tiRNA Detection

tiRNA expression in prostate cancer cell was analyzed using the miRNA qPCR Master Mix Kit (Agilent Technologies, Santa Clara, CA, USA). Briefly, candidate tiRNAs were selected based on previous research results, and their mimics were transfected into prostate cancer cells using Lipofectamine 2000 (Thermo Fisher Scientific, Waltham, MA, USA). To confirm its expression, RNA was extracted, and cDNA was synthesized using the polyadenylation method. PCR was performed according to the miRNA QPCR Master Mix protocols, and universal primers were used as reverse primers in the cDNA synthesis kit. Normalization was applied to the expression of U6, and the 2^−ΔΔCT^ method was used to relatively quantify tiRNA expression levels.

### 2.12. Immunoblot Analysis

Proteins were extracted using a lysis buffer, and the protein concentration was quantified using the Bradford method. Afterward, the denatured proteins were prepared and separated by SDS-PAGE gel electrophoresis based on their size. The separated proteins were transferred to nitrocellulose membranes, and the immunoblots were serially blocked, washed, and incubated with the appropriate primary and secondary antibodies for antigen-antibody interactions. Immunoblot intensity was measured using a ChemiDoc EQ system (Bio-Rad, Hercules, CA, USA). Primary antibodies used in this study are listed in Supplementary Table S1.

### 2.13. Statistical Analysis

All data were subjected to analysis of variance pursuant to the general linear model (PROC-GLM) in the SAS statistical environment (SAS Institute, Cary, NC, USA). All the data were analyzed by one-way ANOVA with the corresponding Tukey’s post-hoc test. All the quantitative results confirmed that there were significant differential effects on each cell type in response to the treatment. Differences with a probability value of *p* < 0.05 were considered statistically significant. Significance levels are indicated by asterisks or numbers (* *p* < 0.05, ** *p* < 0.01, and *** *p* < 0.001) (^#^ *p* < 0.05, ^##^ *p* < 0.01, and ^###^ *p* < 0.001). Data are presented as mean ± standard error of the mean unless otherwise stated.

## 3. Results

### 3.1. Osthole Inhibited Cell Viability and Cell Growth in PC3 and DU145 Prostate Cancer Cells

**Figure 1:**
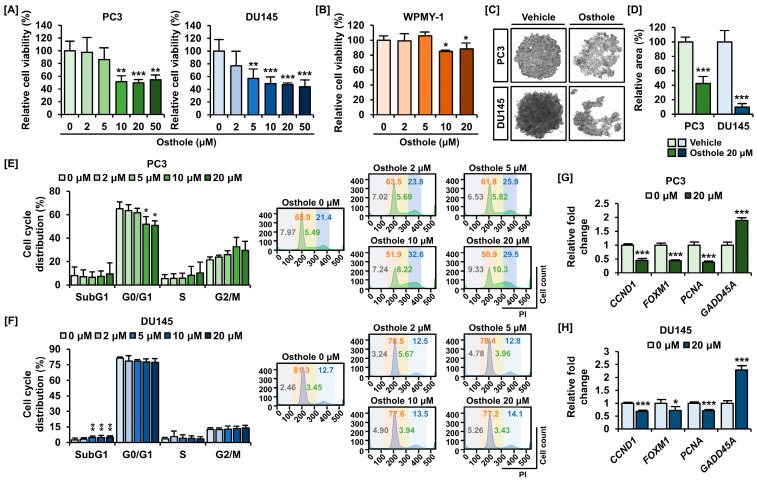
Osthole inhibited cell viability and cell growth in prostate cancer cell lines. (**A**) Anti-viability effect of osthole in PC3 and DU145 cells. (**B**) Viability did not change with osthole in normal prostate cells, WPMY-1. (**C**) Unorganized spheroid formation by osthole (20 μM) was observed via the DM3000 microscope. (**D**) The relative areas of spheroids were quantified using Image J. (**E**,**F**) The cell cycle distribution was changed by osthole, indicating cell cycle arrest in prostate cancer cells. (**G**,**H**) mRNA expression of cell growth-related genes in prostate cancer cell regulated by the osthole. Asterisks indicate the significance level between vehicle and treatment group (* *p* < 0.05, ** *p* < 0.01, and *** *p* < 0.001).

First, we investigated the effects of osthole on the viability and growth of prostate cancer cells. When osthole was added up to 20 μM, the cell viability of prostate cancer was decreased to 49.8% in PC3 cells and 47.3% in DU145 cells (Figure 1A). Accordingly, we selected 20 μM of osthole as the optimal concentration. Moreover, we clarified whether osthole was toxic to normal cells using WPMY-1, a normal prostate cell line, and observed only an 11.5% decrease in cellular viability, suggesting that osthole specifically targets cancer cells (Figure 1B). Furthermore, osthole caused prostate cancer cells to aggregate and grow as spheroids, implying that it might effectively disrupt cellular interactions in vivo (Figure 1C). In PC3 cells, spheroid formation was reduced by 42.3% upon osthole treatment, whereas it was reduced by one-tenth in DU145 cells (Figure 1D). In addition, we performed PI staining to measure cell cycle progression in PC3 and DU145 cells. Osthole increased the sub-G1 phase in both cell lines and elevated the G2/M population, with less of an effect observed in DU145 cells (Figure 1E,F). Additionally, the mRNA expression of the cell cycle-related factors *CCND1*, *FOXM1*, and *PCNA* mRNA expressions were significantly decreased in both osthole-treated PC3 and DU145 cells, while that of *GADD45A*, a DNA damage marker, was increased (Figure 1G,H). These results indicated that osthole influences the viability and growth of prostate cancer cells.

### 3.2. Osthole Induced Disruption of Mitochondria Membrane Potential (ΔΨm) and Increased Reactive Oxygen Species Generation in Prostate Cancer Cells

**Figure 2:**
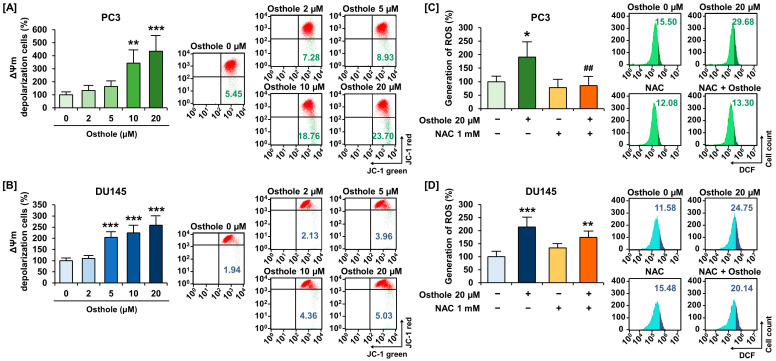
Osthole induced depolarization of mitochondria membrane and ROS generation in PC3 and DU145 cells. (**A**,**B**) Mitochondria membrane potential (ΔΨm) in prostate cancer was measured through relative ratio of JC-1 green by osthole for 48 h. (**C**,**D**) 20 μM of osthole and 1 mM of NAC, the radical scavenger, were co-treated for 48 h, and the relative percentages of the DCF ratio were calculated. Asterisks indicate the significance level between vehicle and treatment group (* *p* < 0.05, ** *p* < 0.01, and *** *p* < 0.001). A crosshatch (#) mark indicates the significance levels between osthole treatment and the osthole with NAC treatment group (^##^ *p* < 0.01).

We investigated the effects of osthole on mitochondrial function in prostate cancer cell lines, focusing on the depolarization of ΔΨm and generation of ROS. Osthole significantly increased the ΔΨm depolarization ratio up to 435% in PC3 cells and up to 259% in DU145 cells (Figure 2A,B). In addition, 20 μM of osthole increased ROS generation up to 191% in PC3 and up to 213% in DU145 cells compared to the vehicle (Figure 2C,D). Moreover, osthole-induced ROS generation was ameliorated to a normal state by NAC, a free radical scavenger, in PC3 cells (Figure 2C), whereas co-treatment of NAC suppressed the osthole-induced ROS generation from 214% to 174% in DU145 cells, but not significantly (Figure 2D). Accordingly, we concluded that osthole induces mitochondrial dysfunction and oxidative stress in prostate cancer cells.

### 3.3. Osthole Interfered with ER-Mediated Calcium Homeostasis in Prostate Cancer Cells

**Figure 3:**
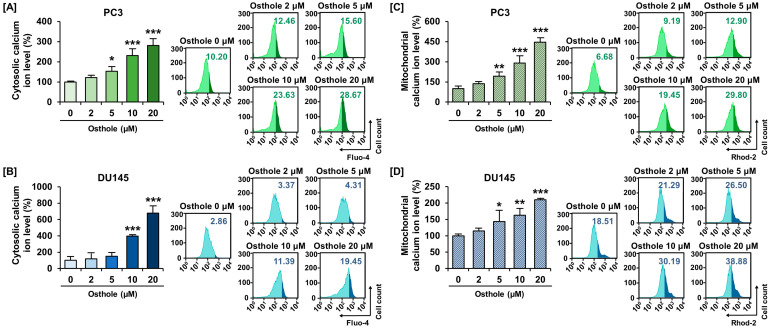
Osthole disrupted calcium homeostasis in prostate cancer cell lines. (**A**,**B**) The cytosolic calcium ion level was upregulated by osthole in PC3 and DU145. The Flou-4AM was used to detect the cytosolic calcium ion. (**C**,**D**) The mitochondrial calcium ion upregulation in prostate cancer cells by osthole was detected using Rhod-2 AM. These results indicated the calcium ions overload in each organelle by the osthole. Asterisks indicate the significance level between vehicle and treatment group (* *p* < 0.05, ** *p* < 0.01, and *** *p* < 0.001).

**Figure 4:**
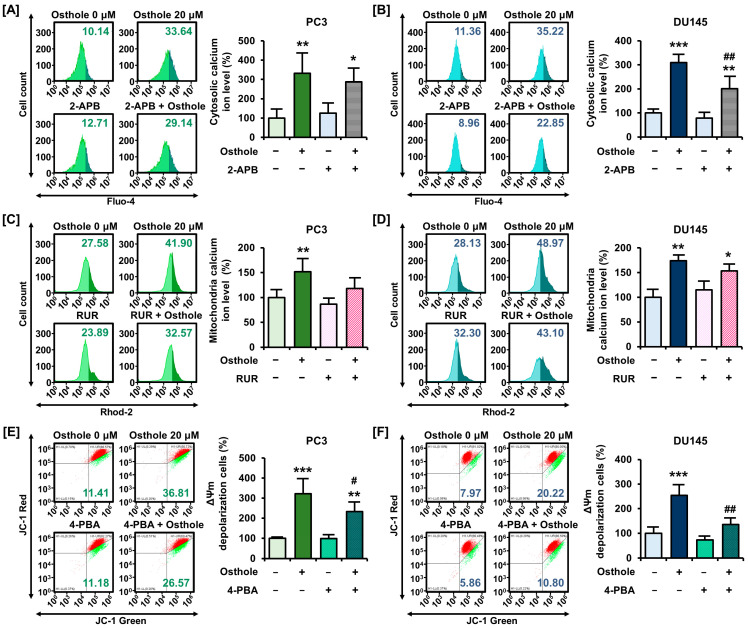

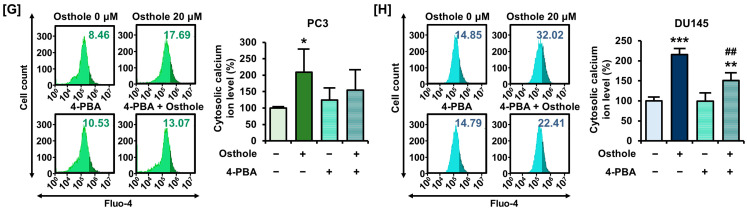
Alleviation of osthole-induced calcium ion accumulation by calcium channel inhibitors and ER stress inhibitor in PC3 and DU145 cells. (**A**,**B**) 2-APB (5 μM) as IP_3_R antagonist and osthole (20 μM) were co-treated for 24 h and stained to Fluo-4 dye to detect cytosolic calcium levels in PC3 and DU145 cells. (**C**,**D**) 8 μM of RUR and osthole (20 μM) were co-treated for 48 h to detect the mitochondrial calcium level in PC3 and DU145 cells. (**E**,**F**) As a selective ER stress inhibitor, 0.1 mM of 4-PBA were co-treated with osthole and ΔΨm were measured by JC-1 dye in prostate cancer cells. (**G**,**H**) Changes of cytosolic calcium levels by co-treatment of 4-PBA and osthole in prostate cancer cells. Asterisks indicate the significance level between vehicle and treatment group (* *p* < 0.05, ** *p* < 0.01, and *** *p* < 0.001). Crosshatch (#) marks indicate the significant levels between osthole treatment and the other co-treatment group (^#^ *p* < 0.05 and ^##^ *p* < 0.01).

The regulation of intracellular calcium homeostasis is one of the functions of mitochondria. Therefore, we used Fluo-4 AM and Rhod-2 AM dyes to determine whether osthole disrupts calcium homeostasis in the cytoplasm and mitochondrial matrix of prostate cancer cells, respectively. Osthole increased cytosolic calcium ion levels by up to 281% in PC3 and 679% in DU145 cells compared to the vehicle (Figure 3A,B). Furthermore, mitochondrial calcium ion levels increased by up to 446% in PC3 and 210% in DU145 cells treated with osthole (Figure 3C,D). Moreover, to trace the suspected calcium ion flow and figure out causality, we utilized 2-aminoethoxydiphenyl borate (2-APB) targeting inositol-1,4,5-triphosphate receptors (IP_3_R) in endoplasmic reticulum (ER), RUR targeting the mitochondrial calcium uniporter (MCU), and 4-phenylbutyric acid (4-PBA) as a selective ER stress inhibitor [20,21]. Co-treatment of 2-APB or RUR with osthole seemed to decrease the cytosolic calcium accumulation from 331% to 287% and mitochondrial calcium accumulation from 151% to 118% in PC3 cells; however, these changes were not statistically significant (Figure 4A,C). Similarly, cytosolic calcium upregulation by osthole was alleviated by 2-APB from 310% to 210% in DU145 cells (Figure 4B). Furthermore, the mitochondrial calcium level was also decreased by RUR, but not significantly (Figure 4D). Additionally, we progressed conditional treatment with 4-PBA and osthole and measured variation in ΔΨm and cytosolic calcium levels in each cell line. As a result, 4-PBA co-treatment with osthole mitigated the ΔΨm depolarization ratio from 322% to 232% in PC3 cells and from 253% to 135% in DU145 cells, significantly (Figure 4E,F). Additionally, cytosolic calcium levels decreased from 209% to 154% in PC3 cells and from 215% to 150% in DU145 cells (Figure 4G,H). These results suggested that osthole induces ER stress and calcium ion release from the ER into the cytoplasm, which may affect mitochondrial permeabilization.

### 3.4. Osthole Impeded Mitochondria Respiration in Prostate Cancer Cells

**Figure 5:**
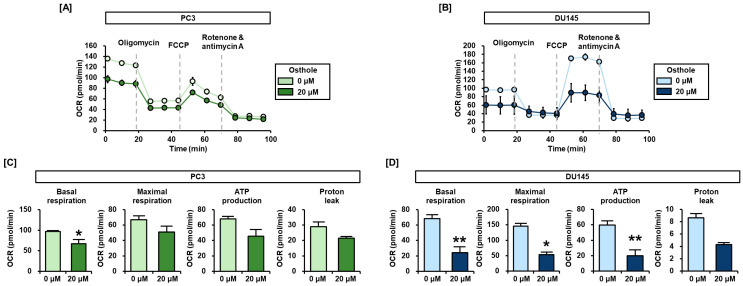
Mitochondrial respiration was impeded by osthole in prostate cancer cell lines. (**A**,**B**) Changes in real-time oxygen consumption rate (OCR) in response to oligomycin, FCCP, rotenone/antimycin A, and osthole treatment were detected in PC3 and DU145 cells using an XFe analyzer. (**C**,**D**) Mitochondrial respiration parameters that could be obtained from the above OCR graphs were indicated as bar graphs. Asterisks indicate the significance level between vehicle and treatment group (* *p* < 0.05 and ** *p* < 0.01).

Afterward, considering our previous results on mitochondrial depolarization, we investigated ATP generation in the mitochondrial respiratory chain using a Seahorse XFe analyzer. To measure mitochondrial respiration in prostate cancer cells, oligomycin, FCCP, rotenone, and antimycin A were serially and periodically injected into the cells (Figure 5A,B). With 20 μM of osthole treatment, the basal respirations were decreased by 31% compared to the vehicle group in PC3 cells (Figure 5C). In addition, other respiration parameters such as maximal respiration, ATP production, and proton leakage were reduced by 24%, 33%, and 26%, respectively, in PC3 (Figure 5C). In the case of osthole-treated DU145 cells, respiratory parameters were significantly reduced by approximately 60–70% (Figure 5D). Accordingly, we confirmed that osthole affects mitochondrial depolarization and respiration in prostate cancer cells.

### 3.5. Osthole Induced Mitochondrial Dysfunction via tiRNA^HisGTG^ Regulation

**Figure 6:**
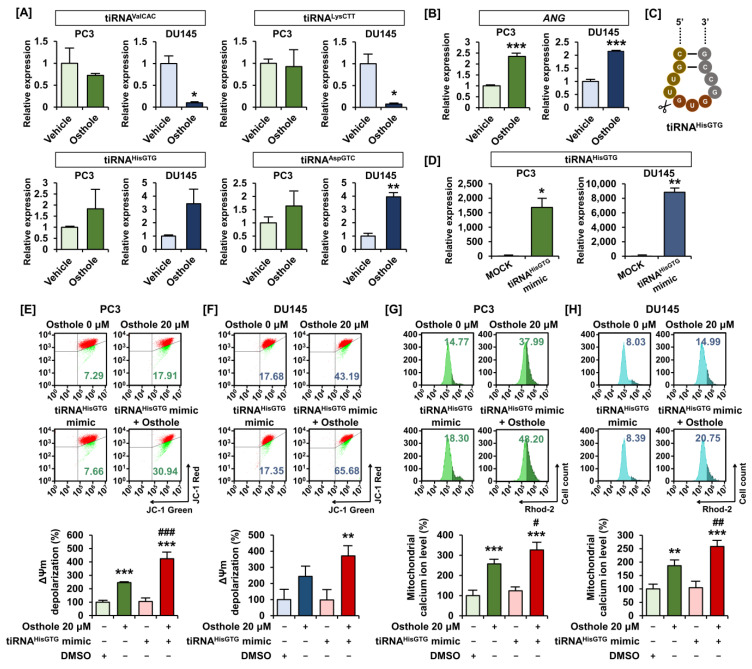
Regulation of tiRNA^HisGTG^ expressions by osthole in PC3 and DU145 cells. (**A**) The change in expression of tiRNA^ValCAC^, tiRNA^LysCTT^, tiRNA^HisGTG^, and tiRNA^AspGTC^ in response to osthole (20 μM) on PC3 and DU145 cells was confirmed using real-time PCR. (**B**) The relative mRNA levels change of *angiogenin* (*ANG*) by osthole in prostate cancer cells was examined using real-time PCR. (**C**) Schematic image about the sequences and cleavage positions in the anticodon loops of tiRNA^HisGTG^. (**D**) Confirmation of tiRNA^HisGTG^ mimic transfection to PC3 and DU145 cells. Transfections were conducted for 5 h. (**E**,**F**) ΔΨm changes by transfection of tiRNA^HisGTG^ mimic and co-treatment with osthole for 48 h in prostate cancer cells. (**G**,**H**) Mitochondrial calcium ion level changes by transfection of tiRNA^HisGTG^ mimic and osthole treatment for 48 h. Asterisks indicate the significance level between vehicle and treatment group (* *p* < 0.05, ** *p* < 0.01, and *** *p* < 0.001). Crosshatch (#) marks indicate the significant levels between osthole and tiRNA treatment group (^#^ *p* < 0.05, ^##^ *p* < 0.01, and ^###^ *p* < 0.001).

Based on previous studies on the physiological function of tiRNAs in cancer cells, we selected four candidate tiRNAs (tiRNA^ValCAC^, tiRNA^LysCTT^, tiRNA^HisGTG^, and tiRNA^AspGTC^) to confirm their expression in prostate cancer cells (Figure 6A). Under stressful conditions, angiogenin cleaves the anticodon loops of tRNA and produces tiRNAs. Upon osthole treatment, *ANG* mRNA expression dramatically increased in both PC3 and DU145 cells (Figure 6B). Considering our results and those of previous studies, we selected tiRNA^HisGTG^ as a possible osthole-induced therapeutic target in prostate cancer cells (Figure 6C). Then, we transfected the tiRNA^HisGTG^ mimic into each prostate cancer cell line and confirmed its overexpression using quantitative PCR (Figure 6D). Using this tiRNA^HisGTG^ mimic, we investigated how it affects the osthole-induced ΔΨm depolarization and mitochondrial calcium accumulation. Overexpression of tiRNA^HisGTG^ made depolarization of ΔΨm more severe compared to sole treatment of osthole, from 245% to 424% in PC3 and 244% to 371% in DU145 cells (Figure 6E,F). Similarly, mitochondrial calcium levels significantly increased from 257% to 326% in PC3 and from 186% to 258% in DU145 cells following tiRNA^HisGTG^ overexpression (Figure 6G,H). Accordingly, we confirmed that osthole regulates tiRNA^HisGTG^ expression in prostate cancer cells, inducing mitochondrial dysfunction.

### 3.6. Osthole Regulated the PI3K or MAPK Pathways That Are Associated with Cell Survival in Prostate Cancer Cells

**Figure 7:**
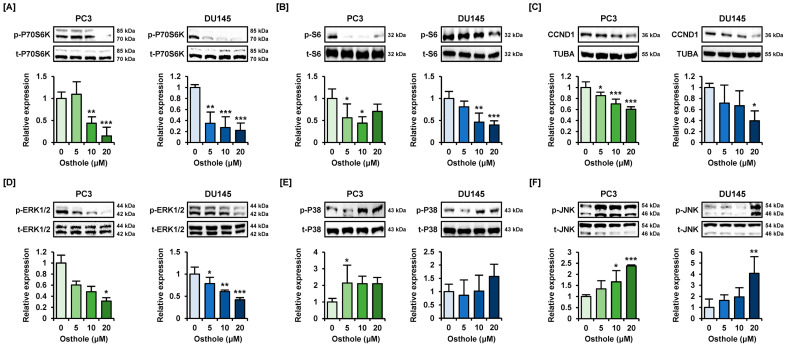
Osthole regulated the PI3K and MAPK pathways in PC3 and DU145 cells. (**A**–**F**) Immunoblots about phosphorylation of P70S6K (**A**), S6 (**B**), CCND1 (**C**), ERK1/2 (**D**), P38 (**E**), and JNK (**F**) by osthole treatment for 24 h. Additionally, its relative expressions were normalized by each total protein or alpha-tubulin (TUBA). Asterisks indicate significant levels of between naïve and osthole-treated cells. Asterisks indicate the significance level between naïve and treatment groups (* *p* < 0.05, ** *p* < 0.01, and *** *p* < 0.001).

We treated prostate cancer cells with osthole for 24 h to determine whether it affects intracellular signaling mechanisms (Figure 7). The phosphorylation of P70S6K sharply decreased by approximately one-fifth in naïve cells in response to osthole treatment in both prostate cancer cell lines (Figure 7A). Additionally, phosphor-S6 and total-CCND1 protein expressions were reduced by osthole to almost half of that in the naïve group (Figure 7B,C). ERK1/2 phosphorylation also showed a gradual reduction in response to osthole treatment in a dose-dependent manner in both PC3 and DU145 cells (Figure 7D). Contrastingly, P38 and JNK MAPK phosphorylation levels were upregulated by 1.5-fold to four-fold by osthole in each prostate cancer cell line (Figure 7E,F). These results confirm that osthole regulates cell survival-related PI3K and MAPK and cell cycle-related CCND1 in PC3 and DU145 cells.

### 3.7. Osthole Activated ER Stress and Autophagy Pathway in Prostate Cancer Cells

**Figure 8:**
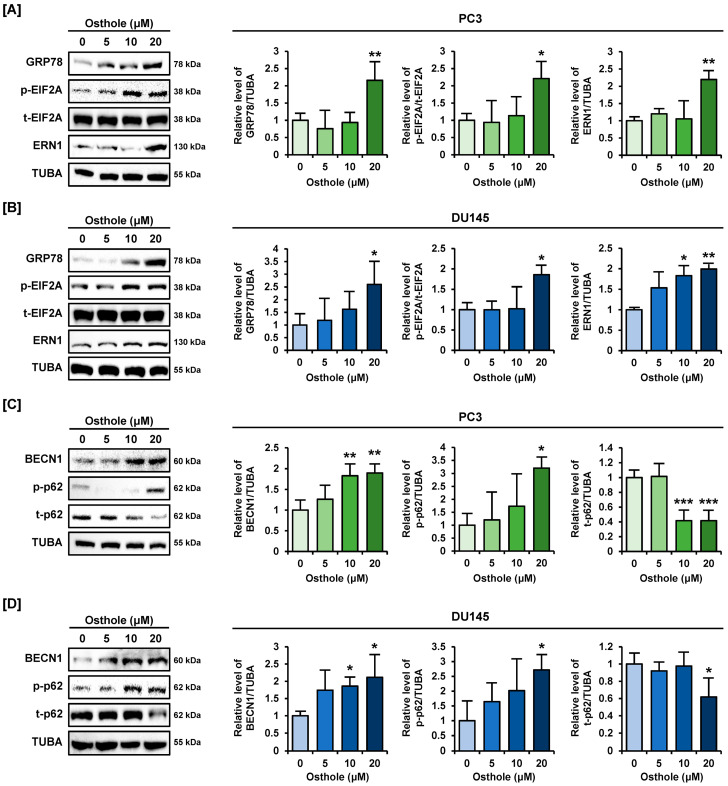
ER stress and autophagy pathway were activated by osthole in prostate cancer cells. (**A**,**B**) Immunoblots about ER stress proteins (GRP78, EIF2A, and ERN1) and their expressions upon osthole treatment for 24 h in prostate cancer cells. (**C**,**D**) Immunoblots related to autophagy proteins (BECN1, phospho-p62, and total-p62) and their expressions by osthole treatment for 24 h in prostate cancer cells. The above results indicate regulation of ER stress and autophagy by osthole in prostate cancer. Other proteins were normalized using TUBA. Asterisks indicate the significance level between naïve and treatment groups (* *p* < 0.05, ** *p* < 0.01, and *** *p* < 0.001).

Furthermore, to confirm whether osthole induces ER stress signaling, as confirmed in our previous experiments, we investigated ER stress via western blot analysis. In PC3 cells, GRP78, phosphor-EIF2A, and ERN1 expressions were significantly increased over two-fold by 20 μM of osthole (Figure 8A). Similarly, ER stress-related protein expression was upregulated two-fold to 2.6-fold by osthole in DU145 cells (Figure 8B). In addition, when autophagy-related proteins were treated with osthole, the expression of BECN1 and phosphorylation of Ser^349^ residue p62 protein increased approximately two-fold to three-fold in both PC3 and DU145 cell lines (Figure 8C,D). Contrastingly, total-p62 proteins levels gradually decreased to 42% and 62%, respectively, compared to the control group, in osthole-treated PC3 and DU145 cells (Figure 8C,D). These results indicate that p62 protein is degraded and autophagy signals are activated by osthole in prostate cancer cells. Therefore, our results demonstrated that osthole induces ER stress and autophagy in prostate cancer cells.

## 4. Discussion

This study demonstrated that osthole specifically inhibited the viability of prostate cancer cells but did not affect normal prostate cells. In addition, osthole suppressed spheroid formation, suggesting its potential in vivo. Cyclin D1 is a well-known cell cycle regulator involved in the G1-S phase transition along with other cyclin-dependent kinases [22]. FOXM1 also participates in the progression into the S and M phases and regulates the expression of other genes during each cell phase transition [23]. PCNA is expressed during DNA replication and repair processes that regulate the cell cycle, and GADD45A is a typical DNA damage marker. Therefore, osthole induces G2/M cell cycle arrest and DNA damage, leading to inhibition of prostate cancer cell growth. Recent studies have shown that cell cycle arrest is also induced in the G2/M phase by ER stress [24,25]. ER stress inhibits cyclin D1 translation, resulting in the loss of cyclin D1 and G1 cell cycle arrest [26].

The ER is a large and dynamic organelle that plays a role in protein synthesis, transport, carbohydrate metabolism, and calcium storage. Moreover, the ER covers approximately 20% of the mitochondrial surface so that both organelles conduct key intracellular functions together, such as regulating cell survival, energy metabolism, and calcium homeostasis [27,28,29]. First, in the aspect of calcium homeostasis, there are ryanodine receptors (RyR), IP_3_R, and sarcoendoplasmic reticulum calcium ATPase in the ER membrane. In addition, mitochondrial calcium ion uptake is mediated by the MCU, rapid mode of uptake (RaM), and mitochondrial RyR (mRyR) [30]. To determine the mechanism of how calcium flow through the ER and mitochondria is changed by osthole, we used 2-APB (IP_3_R inhibitor), RUR (MCU inhibitor), and 4-PBA (ER stress inhibitor) [31,32,33]. RUR did not alleviate mitochondrial calcium uptake, which implied that the other types of calcium channels, such as the non-MCU mRyR, might be involved in both PC3 and DU145 cells [33]. In our results where 2-APB and 4-PBA significantly blocked calcium flux in DU145, it is assumed that calcium release from IP_3_R due to ER stress by osthole is the main cause of mitochondrial dysfunction in DU145 cells. However, in the case of PC3, the blocking of calcium flux through 2-APB and RUR was not significant, which is presumed to be because ER stress is not significantly suppressed by 4-PBA. Additionally, there is a possibility that calcium released due to stress may have fluxed through RyR or other calcium channels, not only to IP_3_R [33,34].

Second, prolonged ER stress affects the redox environment and modulates ROS production [35]. In normal states, calcium ions released from the ER are taken up by the mitochondria, where they stimulate mitochondrial respiration and ATP production [36]. However, under excessive calcium accumulation in mitochondria with oxidative stress, complex I and III of the mitochondrial respiratory chain could be inhibited and further increase ROS production [37,38]. Moreover, ROS, as one of the major causes of oxidative protein folding in ER, could make ER more susceptible to oxidative stress by limiting antioxidant defense system [35]. Through our results, osthole-induced ROS and ER stress causes dysregulation of calcium homeostasis with mitochondrial dysfunction, which further leads to ATP deletion in the OXPHOS system [39].

Likewise, under various stress conditions such as oxidative stress or ER stress, tiRNAs are generated from pre-tRNAs and mature tRNAs via cleavage by Dicer or angiogenin. Plus, tiRNAs play important physiological roles. For instance, tiRNAs could regulate the PI3K pathway to suppress the proliferation of cancer cells [40] and could also regulate autophagy signaling, further affecting cancer progression [41]. The tiRNA^HisGTG^ discovered in this study correlated with cell survival and death by synergizing with natural products in colon cancer [42,43]. In particular, tiRNA-5, which tiRNA^HisGTG^ belongs to, is deeply associated with stress, and this tiRNA profile could reflect the degree of oxidative stress in specific diseases [44]. Therefore, while much remains to be seen, it is possible that tiRNAs may also affect mitochondrial dysfunction [45]. This study confirmed that the expression of tiRNA^HisGTG^ was increased by osthole and demonstrated that it synergizes with osthole to induce mitochondrial dysfunction. Further studies are required to elucidate the detailed function of this tiRNA.

PI3K and MAPK are the key regulators of cell survival. PI3K/AKT is upregulated in patients with prostate cancer and is being investigated as a therapeutic target for castration-resistant prostate cancer [46,47]. In addition, inhibition of the PI3K/AKT pathway effectively suppresses cell proliferation and metastasis in prostate cancer cells [48]. Similar, ERK expression is also known to be one of the majors signaling networks involved in advanced prostate cancer [49]. Likewise, osthole suppress the survival-related pathway in prostate cancer cells, whereas it upregulates the P38MAPK and JNK signals. These kinds of MAPK, P38MAPK and JNK, are well-known kinases that are activated by oxidative stress [50,51]. Those oxidative stress-mediated P38MAPK and JNK could inhibit the proliferation and migration of prostate cancer stem cells. Further, P38MAPK and JNK can regulate the balance between apoptosis and autophagy by external stress [52]. During endurable-stress conditions, autophagy could be activated as a survival mechanism for cancer cells. However, the pro-apoptotic function of autophagy can be induced by ER stress and the downregulation of PI3K [53,54]. Previous studies have shown that FGF21 promotes autophagy by inhibiting the PI3K/AKT/mTOR signaling pathway in prostate cancer cells [55]. Similarly, ER stress-activated ERN1 induces the phosphorylation of AMPK, indicating the induction of autophagy [56,57]. Consistent with the results of a previous study, osthole increased the expression of Beclin1 and phosphorylation of p62, which indicate the formation of autophagosome, and decreased total-p62 expression, meaning degradation to autolysosome in prostate cancer cells.

In this study, we demonstrated the anti-viability effects of osthole on prostate cancer cells by focusing on mitochondrial function and calcium ion regulation. In particular, osthole upregulated tiRNA^HisGTG^ expression, thereby depolarizing the mitochondrial membrane and causing cytosolic calcium accumulation to inhibit prostate cancer growth. We also showed that osthole generates excessive ROS and inhibits mitochondrial ROS generation. Osthole also inhibited the signaling of PI3K, which is associated with cell proliferation, and induced autophagic signaling, which can change the fate of cancer cells to death. Therefore, we demonstrated that osthole inhibits prostate cancer cell growth in a non-hormonal manner using two cell lines (PC3 and DU145) with low responsiveness to androgens, paving the way for new therapeutic approaches.

## 5. Conclusions

Here, we found that osthole increases the expression of tiRNA^HisGTG^ in prostate cancer cells, causing mitochondrial dysfunction due to intramitochondrial calcium accumulation and inhibiting cell survival. Our study’s limitation is that we only conducted in vitro research. However, recently, the relationships between small regulatory ncRNAs, such as tRNA fragments, and various diseases have been revealed. tiRNA^HisGTG^ can be considered one of them, and while only a correlation between expression and disease has yet been revealed, this study is meaningful in the sense that it explicates a mechanism of how tiRNA^HisGTG^ affects mitochondrial function. The mechanism of how osthole increases tiRNA^HisGTG^ expression still needs to be studied in the future.

## Data Availability

Data are contained within the article.

## Supplementary Materials

The following supporting information can be downloaded at https://www.mdpi.com/article/10.3390/antiox13060669/s1: Table S1: List of antibodies used in this study.
