# Supplementary material for: Osthole Suppresses Cell Growth of Prostate Cancer by Disrupting Redox Homeostasis, Mitochondrial Function, and Regulation of tiRNAHisGTG
Source: Antioxidants (Basel). 2024 May 30;13(6):669. doi: 10.3390/antiox13060669 (PMC11201130; doi:10.3390/antiox13060669)
Supplement: Supplementary file 1 [file antioxidants-13-00669-s001.zip › antioxidants-3001822-supplementary.pdf]

Supplemental Table S1. List of antibodies used in this study.

Antibody	Cat No.	Sources
p-P70S6K (Thr^{421}/Ser^{424})	9204	Cell Signaling Technology
p-S6 (Ser^{235}/Ser^{236})	2211	Cell Signaling Technology
p-ERK1/2 (Thr^{202}/Tyr^{204})	9101	Cell Signaling Technology
p-P38 (Thr^{180}/Tyr^{182})	4511	Cell Signaling Technology
p-JNK (Thr^{183}/Tyr^{185})	4668	Cell Signaling Technology
P70S6K	9202	Cell Signaling Technology
S6	2217	Cell Signaling Technology
ERK1/2	4695	Cell Signaling Technology
P38	9212	Cell Signaling Technology
JNK	9252	Cell Signaling Technology
CCND1	2922	Cell Signaling Technology
GRP78	sc-13968	Santa Cruz Biotechnology
p-EIF2A (Ser^{51})	3398	Cell Signaling Technology
EIF2A	5324	Cell Signaling Technology
ERN1	3294	Cell Signaling Technology
BECN1	3495	Cell Signaling Technology
p-P62 (Ser^{349})	16177	Cell Signaling Technology
P62	88588	Cell Signaling Technology
TUBA	sc-32293	Santa Cruz Biotechnology
